# Supplementary material for: Metagenomic data reveals type I polyketide synthase distributions across biomes
Source: mSystems. 2023 Jun 5;8(3):e00012-23. doi: 10.1128/msystems.00012-23 (PMC10308959; doi:10.1128/msystems.00012-23)
Supplement: Table S1 — Metagenome metadata. [file msystems.00012-23-s0001.pdf]

Study Name	Metagenome Size (Base Pairs)	Biome	Accession number(s)
Hardwood forest soil microbial communities from Morgan-Monroe State Forest, Indiana, United States	3,204,058,601	Forest / Agriculture Soil	3300032180
Hardwood forest soil microbial communities from Morgan-Monroe State Forest, Indiana, United States	1,885,256,956	Forest / Agriculture Soil	3300032205
Hardwood forest soil microbial communities from Morgan-Monroe State Forest, Indiana, United States	1,298,045,371	Forest / Agriculture Soil	3300031715
Hardwood forest soil microbial communities from Morgan-Monroe State Forest, Indiana, United States	1,459,061,319	Forest / Agriculture Soil	3300031754
Hardwood forest soil microbial communities from Morgan-Monroe State Forest, Indiana, United States	1,474,821,223	Forest / Agriculture Soil	3300031718
Soil microbial communities from agricultural site in Penn Yan, New York, United States	1,390,993,318	Forest / Agriculture Soil	3300033551
Soil microbial communities from agricultural site in Penn Yan, New York, United States	1,285,422,822	Forest / Agriculture Soil	3300030336
Soil microbial communities from agricultural site in Penn Yan, New York, United States	1,522,946,930	Forest / Agriculture Soil	3300033550
Forest soil microbial communities from Eldorado National Forest, California, USA	1,084,380,358	Forest / Agriculture Soil	3300035687
Forest soil microbial communities from Eldorado National Forest, California, USA	999,898,556	Forest / Agriculture Soil	3300034268
Forest soil microbial communities from Eldorado National Forest, California, USA	1,071,649,480	Forest / Agriculture Soil	3300035667
Forest soil microbial communities from Barre Woods Harvard Forest LTER site, Petersham, Massachusetts, United States	1,455,646,258	Forest / Agriculture Soil	3300020579
Forest soil microbial communities from Barre Woods Harvard Forest LTER site, Petersham, Massachusetts, United States	1,494,582,847	Forest / Agriculture Soil	3300021171
Forest soil microbial communities from Barre Woods Harvard Forest LTER site, Petersham, Massachusetts, United States	1,558,207,418	Forest / Agriculture Soil	3300021168
Forest soil microbial communities from Barre Woods Harvard Forest LTER site, Petersham, Massachusetts, United States	1,363,538,788	Forest / Agriculture Soil	3300020582
Soil microbial communities from LAMPS site, Iowa State University, Ames, IA, USA	1,347,980,442	Forest / Agriculture Soil	3300037444
Soil microbial communities from LAMPS site, Iowa State University, Ames, IA, USA	1,401,762,085	Forest / Agriculture Soil	3300037529
Soil microbial communities from LAMPS site, Iowa State University, Ames, IA, USA	1,527,241,072	Forest / Agriculture Soil	3300037523
Soil microbial communities from LAMPS site, Iowa State University, Ames, IA, USA	1,490,249,658	Forest / Agriculture Soil	3300037803
Soil microbial communities from Everglades Agricultural Area, Florida, United States	1,179,311,592	Forest / Agriculture Soil	3300036759
Soil microbial communities from Everglades Agricultural Area, Florida, United States	1,305,585,406	Forest / Agriculture Soil	3300036838
Soil microbial communities from Everglades Agricultural Area, Florida, United States	1,152,188,752	Forest / Agriculture Soil	3300036840
Wetland soil microbial communities from Old Woman Creek delta, Ohio, United States	2,115,715,718	Peat Soil	3300033419
Wetland soil microbial communities from Old Woman Creek delta, Ohio, United States	1,217,355,322	Peat Soil	3300033483
Wetland soil microbial communities from Old Woman Creek delta, Ohio, United States	1,690,667,107	Peat Soil	3300033485
Wetland soil microbial communities from Old Woman Creek delta, Ohio, United States	1,546,155,278	Peat Soil	3300033414
Wetland soil microbial communities from Old Woman Creek delta, Ohio, United States	1,099,733,367	Peat Soil	3300033488
Peat permafrost microbial communities from Stordalen Mire near Abisko, Sweden	1,299,338,055	Peat Soil	3300030739
Peat permafrost microbial communities from Stordalen Mire near Abisko, Sweden	1,607,780,293	Peat Soil	3300031261
Peat permafrost microbial communities from Stordalen Mire near Abisko, Sweden	2,638,818,371	Peat Soil	3300030906
Peat permafrost microbial communities from Stordalen Mire near Abisko, Sweden	2,731,153,271	Peat Soil	3300031788
Peat permafrost microbial communities from Stordalen Mire near Abisko, Sweden	1,367,811,048	Peat Soil	3300030019
Arctic peat soil microbial communities from the Barrow Environmental Observatory site, Barrow, Alaska, USA	2,085,521,650	Peat Soil	3300006642, 3300025888, 3300006638, 3300006950
Rhizosphere microbial communities from Carex aquatilis grown in University of Washington, Seatle, WA, United States	1,337,824,726	Peat Soil	3300031344
Rhizosphere microbial communities from Carex aquatilis grown in University of Washington, Seatle, WA, United States	1,456,417,748	Peat Soil	3300028800
Rhizosphere microbial communities from Carex aquatilis grown in University of Washington, Seatle, WA, United States	1,716,094,089	Peat Soil	3300031711, 3300031712
Tropical peat soil microbial communities from peatlands in Loreto, Peru	2,435,655,096	Peat Soil	3300035643, 3300033805, 3300033806, 3300033977
Soil microbial communities from Populus trichocarpa stands in riparian zone in the Pacific Northwest, United States	1,733,731,890	Peat Soil	3300028802, 3300031226, 3300031455
Peat soil microbial communities from wetlands in Alaska, United States	1,390,497,852	Peat Soil	3300034163, 3300034196, 3300035209
Populus rhizosphere microbial communities from soil in Oregon, United States	2,047,615,555	Rhizosphere	3300036401
Populus rhizosphere microbial communities from soil in Oregon, United States	1,243,199,825	Rhizosphere	3300035692
Populus rhizosphere microbial communities from soil in Oregon, United States	1,094,358,263	Rhizosphere	3300035695
Populus rhizosphere microbial communities from soil in Oregon, United States	1,089,247,126	Rhizosphere	3300035725
Populus rhizosphere microbial communities from soil in Oregon, United States	1,597,279,552	Rhizosphere	3300037068
Corn rhizosphere microbial communities from Kellogg Biological Station, Michigan, USA	1,516,673,119	Rhizosphere	3300025917
Corn rhizosphere microbial communities from Kellogg Biological Station, Michigan, USA	1,561,591,177	Rhizosphere	3300025919
Switchgrass rhizosphere microbial communities from Kellogg Biological Station, Michigan, USA	1,567,954,971	Rhizosphere	3300025923
Switchgrass rhizosphere microbial communities from Kellogg Biological Station, Michigan, USA	1,595,709,255	Rhizosphere	3300025931
Switchgrass rhizosphere microbial communities from Kellogg Biological Station, Michigan, USA	2,410,994,453	Rhizosphere	3300005719
Switchgrass rhizosphere microbial communities from Kellogg Biological Station, Michigan, USA	1,861,559,781	Rhizosphere	3300025986
Miscanthus rhizosphere microbial communities from Kellogg Biological Station, Michigan, USA	1,638,079,859	Rhizosphere	3300025926
Miscanthus rhizosphere microbial communities from Kellogg Biological Station, Michigan, USA	1,485,183,434	Rhizosphere	3300025935
Miscanthus rhizosphere microbial communities from Kellogg Biological Station, Michigan, USA	1,136,041,079	Rhizosphere	3300005328
Miscanthus rhizosphere microbial communities from Kellogg Biological Station, Michigan, USA	1,786,668,392	Rhizosphere	3300005338
Rhizosphere microbial communities from Vellozia epidendroides in rupestrian grasslands, the National Park of Serra do Cipo, Br	957,257,553	Rhizosphere	3300021358, 3300021361, 3300021441
Maize rhizosphere microbial communities from greenhouse at UC Davis, California, United States	1,250,286,332	Rhizosphere	3300031903
Maize rhizosphere microbial communities from greenhouse at UC Davis, California, United States	1,611,017,323	Rhizosphere	3300031852
Arabidopsis thaliana rhizosphere microbial communities from the Joint Genome Institute, USA, that affect carbon cycling	967,377,510	Rhizosphere	3300027665, 3300027682, 3300027695, 3300027717, 3300027876
Populus root and rhizosphere microbial communities from Tennessee, USA	1,372,540,525	Rhizosphere	3300027907
Sediment microbial communities from Yellowstone Lake	3,482,940,763	Freshwater Sediment	3300032516
Sediment microbial communities from Yellowstone Lake	1,558,866,242	Freshwater Sediment	3300035688
Sediment microbial communities from Yellowstone Lake	1,301,812,343	Freshwater Sediment	3300031885
Sediment microbial communities from Yellowstone Lake	2,118,061,919	Freshwater Sediment	3300032046
Sediment microbial communities from Loxahatchee National Wildlife Refuge, Florida, United States	1,356,943,436	Freshwater Sediment	3300038410
Sediment microbial communities from Loxahatchee National Wildlife Refuge, Florida, United States	1,274,909,464	Freshwater Sediment	3300038550
Sediment microbial communities from Loxahatchee National Wildlife Refuge, Florida, United States	1,953,865,168	Freshwater Sediment	3300038408
Sediment microbial communities from Loxahatchee National Wildlife Refuge, Florida, United States	1,686,876,318	Freshwater Sediment	3300038455
Sediment microbial communities from Loxahatchee National Wildlife Refuge, Florida, United States	3,028,796,679	Freshwater Sediment	3300038552
Freshwater lake sediment microbial communities from the University of Notre Dame, USA, for methane emissions studies	1,235,723,351	Freshwater Sediment	3300027902
Freshwater lake sediment microbial communities from the University of Notre Dame, USA, for methane emissions studies	1,356,447,832	Freshwater Sediment	3300027900
Freshwater lake sediment microbial communities from the University of Notre Dame, USA, for methane emissions studies	1,474,716,571	Freshwater Sediment	3300027896
Sediment microbial communities from wetlands near Prado wetlands, California, USA	1,906,967,011	Freshwater Sediment	3300037458
Sediment microbial communities from wetlands near Prado wetlands, California, USA	2,119,703,235	Freshwater Sediment	3300037524
Sediment microbial communities from wetlands near Prado wetlands, California, USA	2,289,580,168	Freshwater Sediment	3300037461
Sediment microbial communities from wetlands near Prado wetlands, California, USA	1,646,841,975	Freshwater Sediment	3300037408
Freshwater microbial communities from Lake Tanganyika, Tanzania	2,767,785,519	Freshwater	3300020109, 3300020220
Freshwater microbial communities from Lake Tanganyika, Tanzania	1,302,498,413	Freshwater	3300020222
Freshwater microbial communities from Lake Tanganyika, Tanzania	1,273,692,525	Freshwater	3300020084
Freshwater microbial communities from Lake Tanganyika, Tanzania	1,460,997,943	Freshwater	3300020074
Freshwater microbial communities from Lake Fryxell liftoff mats and glacier meltwater in Antarctica	1,609,619,801	Freshwater	3300009032
Freshwater microbial communities from Lake Fryxell liftoff mats and glacier meltwater in Antarctica	1,606,851,264	Freshwater	3300009084
Freshwater microbial communities from Lake Fryxell liftoff mats and glacier meltwater in Antarctica	1,628,106,102	Freshwater	3300009083
Freshwater microbial communities from Lake Fryxell liftoff mats and glacier meltwater in Antarctica	1,278,739,541	Freshwater	3300007521
Freshwater microbial communities from Lake Bonney liftoff mats and glacier meltwater in Antarctica	1,474,819,565	Freshwater	3300007519
Freshwater microbial communities from Lake Mendota, Madison, Wisconsin, United States	1,326,969,174	Freshwater	3300035666
Freshwater microbial communities from Lake Mendota, Madison, Wisconsin, United States	1,596,426,758	Freshwater	3300036719
Freshwater microbial communities from Lake Mendota, Madison, Wisconsin, United States	2,099,022,913	Freshwater	3300034101, 3300034284
Freshwater microbial communities from Lake Mendota, Madison, Wisconsin, United States	2,061,414,356	Freshwater	3300034272, 3300034280
Freshwater microbial communities from meromictic Lake La Cruz, Castile	3,391,824,087	Freshwater	3300029286, 3300028581, 3300029268
Freshwater microbial communities from meromictic Lake La Cruz, Castile	1,486,589,259	Freshwater	3300027970, 3300028569
Freshwater microbial communities from Lake Lanier, Atlanta, Georgia, United States	4,054,488,353	Freshwater	3300023184, 3300023174, 3300022752, 3300023179
Marine sediment microbial communities from subtidal zone of North Sea	1,535,122,492	Marine Sediment	3300028600
Marine sediment microbial communities from subtidal zone of North Sea	1,029,147,879	Marine Sediment	3300028599
Marine sediment microbial communities off the coast of San Francisco, CA, United States	1,647,702,068	Marine Sediment	3300037489
Marine sediment microbial communities off the coast of San Francisco, CA, United States	2,382,593,820	Marine Sediment	3300038629
Marine sediment microbial communities off the coast of San Francisco, CA, United States	1,117,689,729	Marine Sediment	3300037835
Marine sediment microbial communities off the coast of San Francisco, CA, United States	1,323,119,854	Marine Sediment	3300037521
Marine sediment microbial communities off the coast of San Francisco, CA, United States	1,109,151,697	Marine Sediment	3300037450
Marine sediment microbial communities off the coast of San Francisco, CA, United States	2,555,594,725	Marine Sediment	3300038548
Marine sediment microbial communities off the coast of San Francisco, CA, United States	1,286,123,733	Marine Sediment	3300037459
Marine sediment microbial communities off the coast of San Francisco, CA, United States	1,241,427,685	Marine Sediment	3300037460
Marine sediment microbial communities off the coast of San Francisco, CA, United States	1,687,513,841	Marine Sediment	3300037540
Marine sediment microbial communities off the coast of San Francisco, CA, United States	1,507,997,651	Marine Sediment	3300037245
Marine sediment microbial communities off the coast of San Francisco, CA, United States	1,376,019,208	Marine Sediment	3300037247
Marine sediment microbial communities off the coast of San Francisco, CA, United States	2,233,392,739	Marine Sediment	3300037246, 3300022413
Coastal sediment microbial communities from Delaware Bay, Delaware, United States	1,697,423,932	Marine Sediment	3300032136
Coastal sediment microbial communities from Oude Bieten Haven, Netherlands	801,797,914	Marine Sediment	3300032251
Coastal sediment microbial communities from Maine, United States	1,509,772,824	Marine Sediment	3300033429
Coastal sediment microbial communities from Maine, United States	1,508,653,967	Marine Sediment	3300032272
Coastal sediment microbial communities from Maine, United States	1,396,560,300	Marine Sediment	3300032231
Coastal sediment microbial communities from Maine, United States	1,298,677,993	Marine Sediment	3300032258
Coastal sediment microbial communities from Maine, United States	1,135,204,813	Marine Sediment	3300032259
Coastal sediment microbial communities from Maine, United States	1,016,401,552	Marine Sediment	3300032262

Coastal sediment microbial communities from Maine, United States	1,157,046,311	Marine Sediment	3300032260		
Marine microbial communities from station ALOHA, North Pacific Subtropical Gyre	2,957,439,220	Seawater	3300032820		
Marine microbial communities from station ALOHA, North Pacific Subtropical Gyre	2,217,368,920	Seawater	3300032278		
Marine microbial communities from station ALOHA, North Pacific Subtropical Gyre	1,666,929,232	Seawater	3300032006		
Marine microbial communities from station ALOHA, North Pacific Subtropical Gyre	1,247,814,780	Seawater	3300031785		
Marine microbial communities from western Arctic Ocean	2,003,936,505	Seawater	3300009786, 3300009173		
Marine microbial communities from western Arctic Ocean	1,801,075,760	Seawater	3300009409, 3300009706		
Marine microbial communities from western Arctic Ocean	2,592,698,426	Seawater	3300009420, 3300009526, 3300009705, 3300009785		
Marine microbial communities from western Arctic Ocean	3,100,549,849	Seawater	3300031802, 3300031804, 3300036808		
Seawater microbial communities from Jervis Inlet, British Columbia, Canada	2,779,312,094	Seawater	3300024518, 3300024520, 3300027865, 3300027881		
Seawater microbial communities from Amundsen Gulf, Northwest Territories, Canada	1,727,509,430	Seawater	3300024521, 3300027872, 3300027997		
Seawater microbial communities from Saanich Inlet, British Columbia, Canada	1,587,034,760	Seawater	3300023210, 3300027861, 3300028045		
Seawater microbial communities from eastern tropical North Pacific Ocean	2,882,053,374	Seawater	3300035157, 3300035202, 3300035203, 3300036767		
Seawater microbial communities from, Arabian Sea, Indian Ocean	3,094,561,910	Seawater	3300035204, 3300035205, 3300035206, 3300035250		
Agave microbial communities from Guanajuato, Mexico	1,044,017,924	Host-Associated (Phyllosphere)	3300005562, 3300006020, 3300030499, 3300009144, 3300027809, 3300030505, 3300030497		
Agave microbial communities from Guanajuato, Mexico	1,110,787,892	Host-Associated (Phyllosphere)	3300005661, 3300010395, 3300030515		
Agave microbial communities from Guanajuato, Mexico	738,289,986	Host-Associated (Phyllosphere)	3300027761, 3300030512, 3300030516		
Phyllosphere microbial communities from UC Gill Tract Community Farm, Albany, California, United States	1,300,189,725	Host-Associated (Phyllosphere)	3300031088, 3300031419		
Phyllosphere microbial communities from UC Gill Tract Community Farm, Albany, California, United States	935,592,278	Host-Associated (Phyllosphere)	3300031132, 3300031370, 3300031418		
Phyllosphere microbial communities from UC Gill Tract Community Farm, Albany, California, United States	1,578,246,309	Host-Associated (Phyllosphere)	3300031134, 3300031413		
Phyllosphere microbial communities from UC Gill Tract Community Farm, Albany, California, United States	1,890,101,419	Host-Associated (Phyllosphere)	3300031087, 3300031110, 3300031133		
Phyllosphere microbial communities from UC Gill Tract Community Farm, Albany, California, United States	1,739,354,604	Host-Associated (Phyllosphere)	3300031084, 3300031112, 3300031420		
Phyllosphere microbial communities from UC Gill Tract Community Farm, Albany, California, United States	1,931,146,294	Host-Associated (Phyllosphere)	3300031372, 3300031414, 3300031416, 3300031417		
Host-associated microbial community of the marine sponge Aplysina aerophoba from Gulf of Piran, Adriatic Sea	1,000,911,300	Host-Associated	3300002222, 3300002150, 3300027328, 3300027951		
Host-associated microbial community of the marine sponge Aplysina aerophoba from Gulf of Piran, Adriatic Sea	1,111,906,834	Host-Associated	3300002159, 3300002448, 3300002147, 3300027327, 3300027386		
Host-associated microbial community of the marine sponge Aplysina aerophoba from Gulf of Piran, Adriatic Sea	1,324,284,378	Host-Associated	3300002160, 3300002151, 3300027391, 3300027532		
Marine algal microbial communities from Sidmouth, United Kingdom	1,003,807,699	Host-Associated	3300009417, 3300009439, 3300009446		
Marine algal microbial communities from Sidmouth, United Kingdom	1,031,151,631	Host-Associated	3300009073, 3300009415, 3300009421, 3300027028		
Tube worm associated microbial communities from hydrothermal vent at the East Pacific Rise, Pacific Ocean	1,278,100,677	Host-Associated	3300028026, 3300028029, 3300028042, 3300028534		
Rumen microbial communities from sheep, dairy cows and beef cattle from various locations	2,488,892,422	Host-Associated	3300028805		
Rumen microbial communities from sheep, dairy cows and beef cattle from various locations	1,559,553,778	Host-Associated	3300037453		
