# Supplementary material for: Metagenomic data reveals type I polyketide synthase distributions across biomes
Source: mSystems. 2023 Jun 5;8(3):e00012-23. doi: 10.1128/msystems.00012-23 (PMC10308959; doi:10.1128/msystems.00012-23)
Supplement: Table S2; Figures S1-S15 — Supplemental table and figures. [file msystems.00012-23-s0002.pdf]

Table S1 (attached separately) - Summary table of all metagenomes analyzed using NaPDoS2 . All metagenomes are listed along with the biome type they fall under and the total size of the metagenome (base pairs).

Class	Subclass	All biomes	Forest / Agricultural soil	Peat Soil	Rhizosphere	Marine Sediment	Freshwater Sediment	Host-associated	Seawater	Freshwater
Modular *cis*-AT	No subclass	13186	3381	2539	1790	951	1070	1275	951	1229
	Hybrid KS	6655	1452	1105	1316	907	687	326	318	544
	Loading module	864	215	139	152	129	54	98	21	56
	Olefin synthase	719	135	79	121	98	58	27	99	102
Iterative *cis*-AT	PUFA	7346	460	791	386	2750	899	134	1141	785
	No subclass	1523	264	178	218	204	229	104	204	122
	Enediyne	1044	173	132	153	403	66	12	49	56
	Aromatic	355	140	48	79	4	34	10	7	33
	PTM-type	207	44	39	36	21	14	6	10	37
trans-AT	No subclass	3061	683	625	473	345	113	291	177	354
	Hybrid KS	156	26	35	27	12	10	15	16	15
	Total KSs	35116	6973	5710	4751	5824	3234	2298	2993	3333
Type I FAS		409	93	36	58	10	35	94	45	38

Table S2 - Type I KS hits classified by NaPDoS2. Type I KS domains listed by NaPDoS2 class and subclass across eight biomes, with the total KS hits across all biomes listed in the third column. The total number of Type I KS hits across all classes is listed in the second to last row. Type I FAS hits were not included in these totals and are listed in the last row.

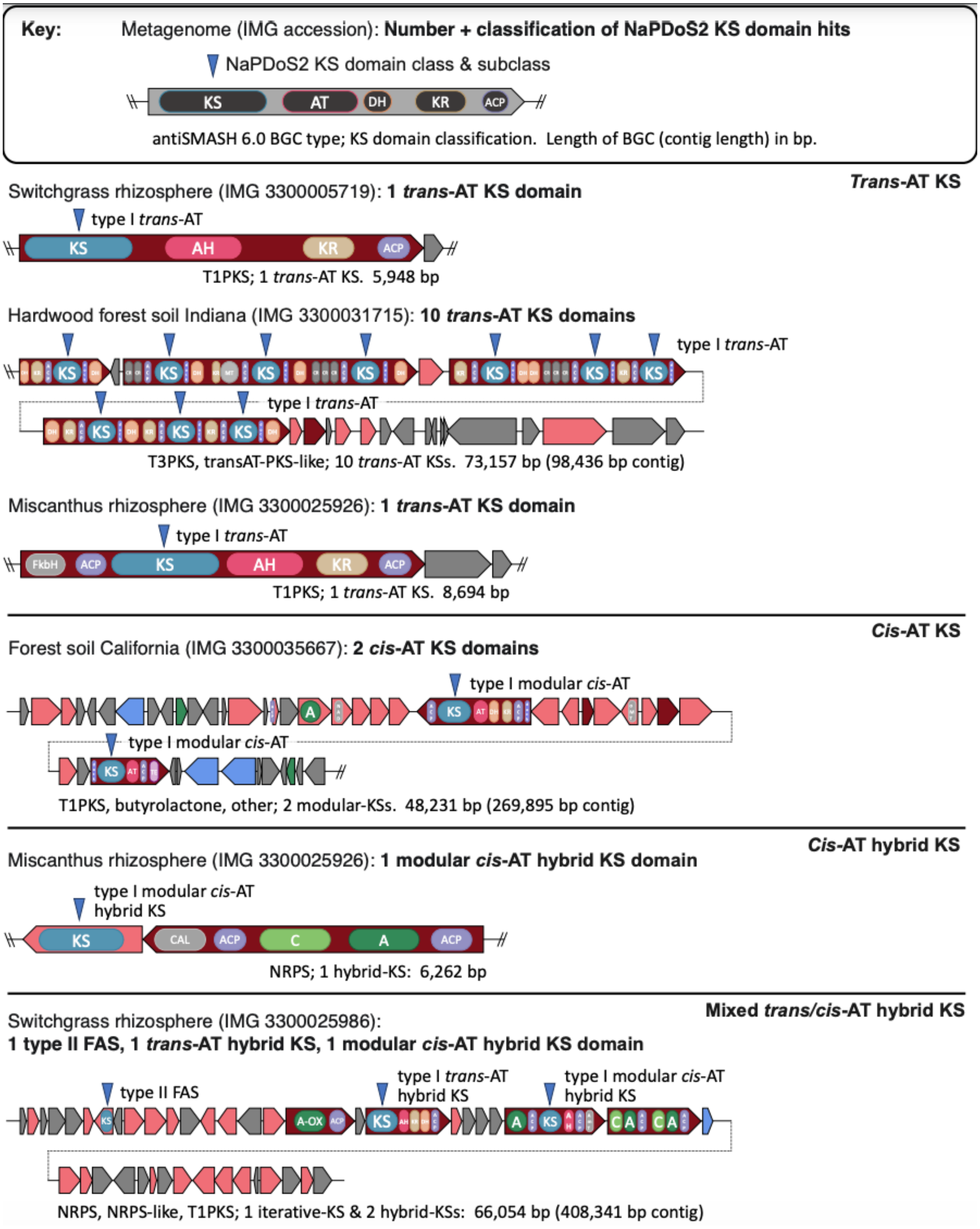
Key: Metagenome (IMG accession): Number + classification of NaPDoS2 KS domain hits
NaPDoS2 KS domain class & subclass
KS
AT
DH
KR
ACP
antiSMASH 6.0 BGC type; KS domain classification. Length of BGC (contig length) in bp.
Trans-AT KS
Switchgrass rhizosphere (IMG 3300005719): 1 trans-AT KS domain
type I trans-AT
KS
AH
KR
ACP
T1PKS; 1 trans-AT KS. 5,948 bp
Hardwood forest soil Indiana (IMG 3300031715): 10 trans-AT KS domains
type I trans-AT
type I trans-AT
T3PKS, transAT-PKS-like; 10 trans-AT KSs. 73,157 bp (98,436 bp contig)
Miscanthus rhizosphere (IMG 3300025926): 1 trans-AT KS domain
type I trans-AT
FkbH
ACP
KS
AH
KR
ACP
T1PKS; 1 trans-AT KS. 8,694 bp
Cis-AT KS
Forest soil California (IMG 3300035667): 2 cis-AT KS domains
type I modular cis-AT
A
KS
type I modular cis-AT
KS
T1PKS, butyrolactone, other; 2 modular-KSs. 48,231 bp (269,895 bp contig)
Cis-AT hybrid KS
Miscanthus rhizosphere (IMG 3300025926): 1 modular cis-AT hybrid KS domain
type I modular cis-AT hybrid KS
KS
CAL
ACP
C
A
ACP
NRPS; 1 hybrid-KS: 6,262 bp
Mixed trans/cis-AT hybrid KS
Switchgrass rhizosphere (IMG 3300025986):
1 type II FAS, 1 trans-AT hybrid KS, 1 modular cis-AT hybrid KS domain
type II FAS
type I trans-AT hybrid KS
type I modular cis-AT hybrid KS
A-OX
ACP
KS
A
KS
C A
C A
NRPS, NRPS-like, T1PKS; 1 iterative-KS & 2 hybrid-KSs: 66,054 bp (408,341 bp contig)

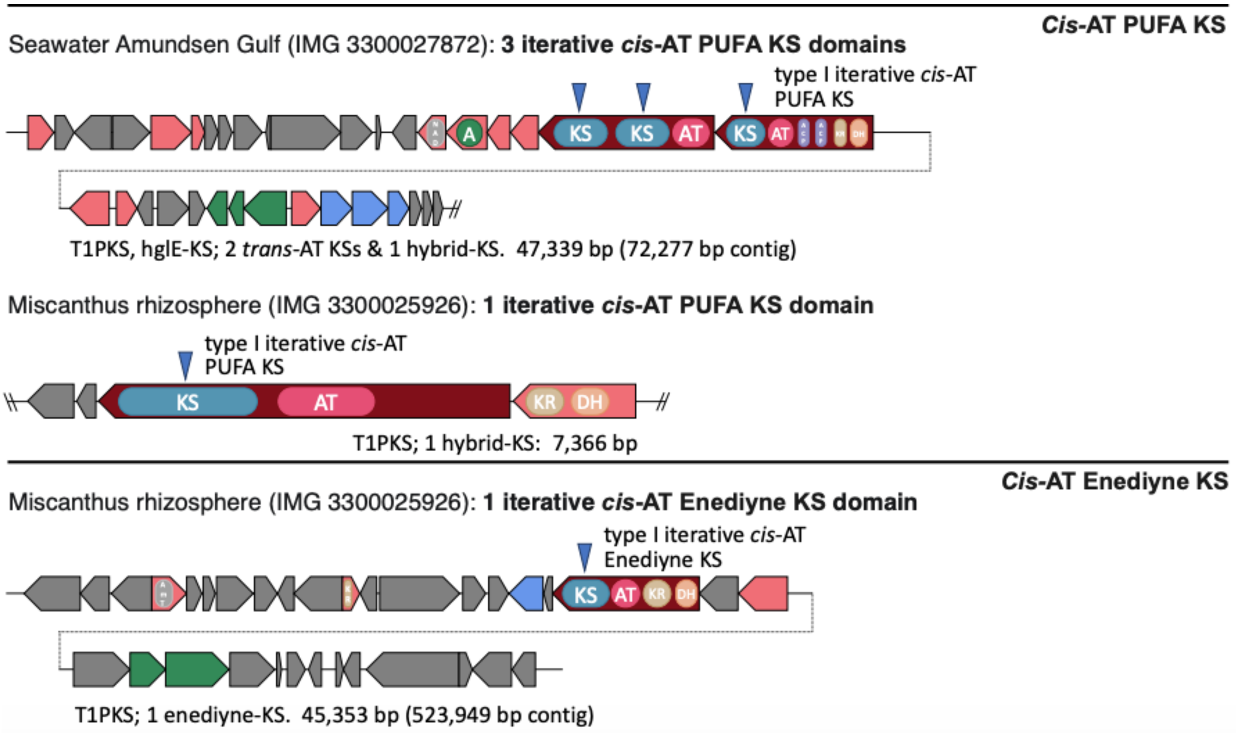

Figure S1 – Validation of NaPDoS2 KS classifications.
The classification of KSs identified by NaPDoS2 was assessed by examining their genomic environment in the metagenomic assembles. They were located using blastP and the JGI IMG interface to query KS domains against their respective metagenome and the scaffold/contig associated with the KS hit extracted and run through antiSMASH 6 to identify the associated BGC. Relevant biosynthetic genes were additionally analyzed using the "transATor" and "PKS/NRPS Analysis Web-site" tools for domain annotation. BGCs were drawn and colored as per antiSMASH 6 (maroon = core biosynthetic gene; pink = additional biosynthetic gene; blue = transport-related genes; green = regulatory genes, gray = other genes). Domain position and function were drawn and colored according to antiSMASH, transATor, and the PKS/NRPS Analysis Website NRPS.IGS (blue = KS = ketosynthase; pink = AH acyl hydrolase, AT = acyl transferase; sand = KR = ketoreductase; pale purple = ACP = Phosphopantetheine acyl carrier protein; orange = DH = dehydratase; dark gray = CR = crotonase; light gray = MT = methyltransferase, CAL = Co-enzyme A ligase domain, NAD = Male sterility protein/3-beta hydroxysteroid dehydrogenase-isomerase family, oMT = oxygen methyltransferase, AmT = aminotransferase; light pink = TE = thioesterase; dark blue = dock = PKS docking C/N terminal or Trans-AT docking domain; light green = C = condensation domain; dark green = A = adenylation domain; A-OX = adenylation domain with integrated oxidase). Blue arrows point to KS hits that NaPDoS2 detected and classified from each metagenome shown in their BGC context; arrows are labeled with their NaPDoS2 classification.

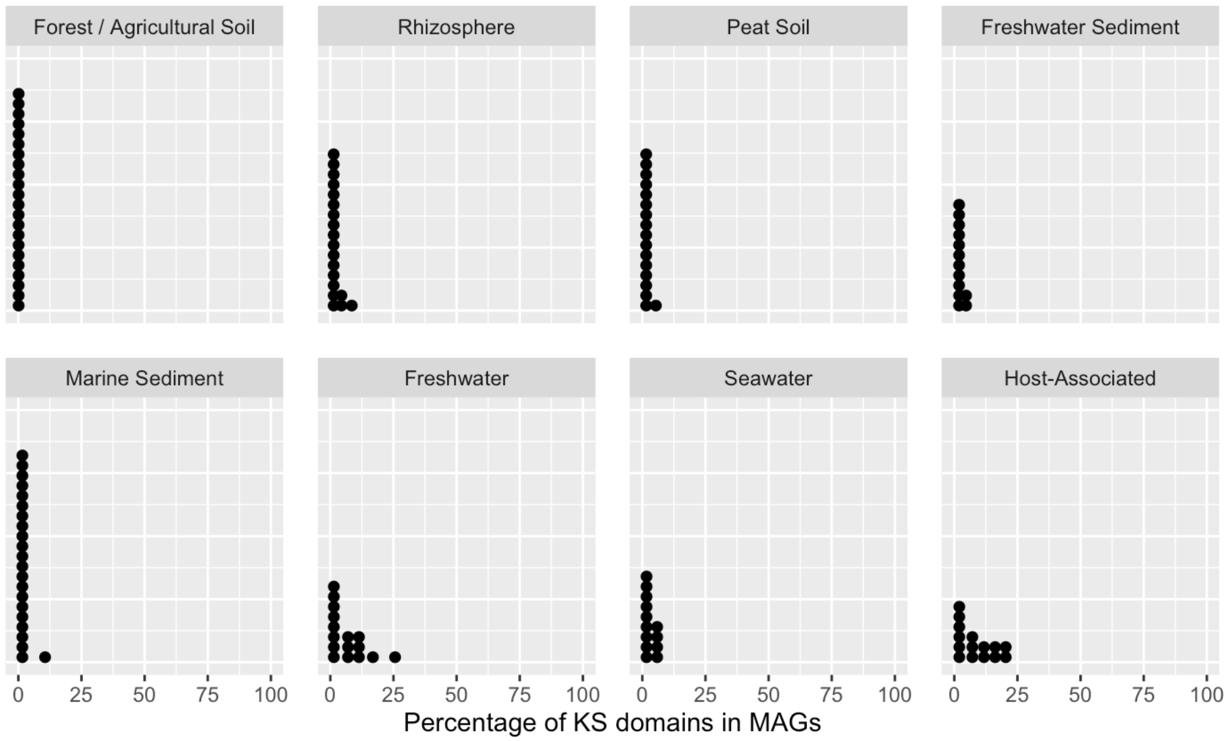

Figure S2 - Percentage of metagenomic KS domains detected within MAGs. Individual metagenomes (137 in total from IMG/M) are represented by black circles. MAGs were binned for each metagenome using MetaBAT according to the JGI IMG automated pipeline. The number of KS domains in each metagenome and MAG was determined using NaPDoS2 and the percentage of metagenomic KSs occurring in MAGs plotted for each metagenome.

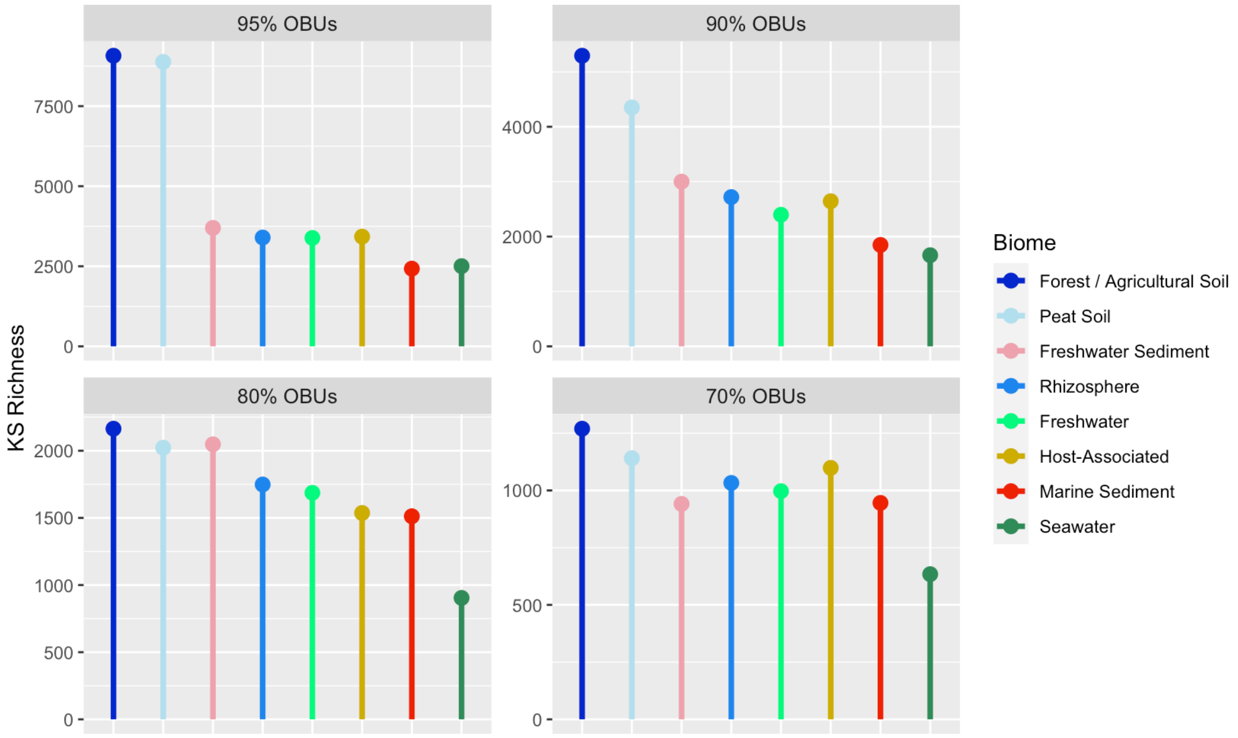

Figure S3 - KS richness across biomes. Bar plot showing Chao1 KS richness across eight biomes. For each biome, 580 full-length KS domains were randomly selected and clustered into Operational Biosynthetic Units (OBUs) at four thresholds ranging from 70% to 95% amino acid sequence identity. The average of 10 analyses per biome is plotted.

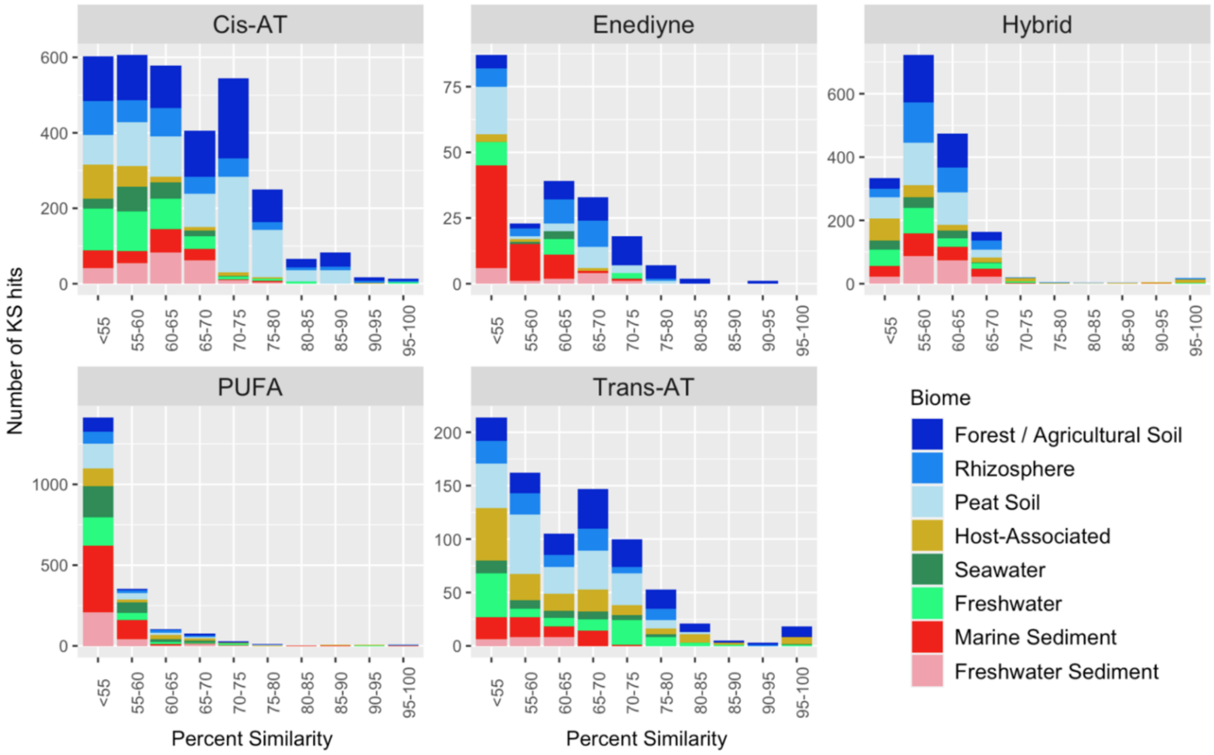

Figure S4 - Percent similarity of metagenomic KSs with their closest MIBiG 2.0 match. Stacked bar charts indicate the biome-level abundance (y-axis) of full-length metagenome-extracted KS domains based on percent similarity with their closest MIBiG 2.0 database match (x-axis).

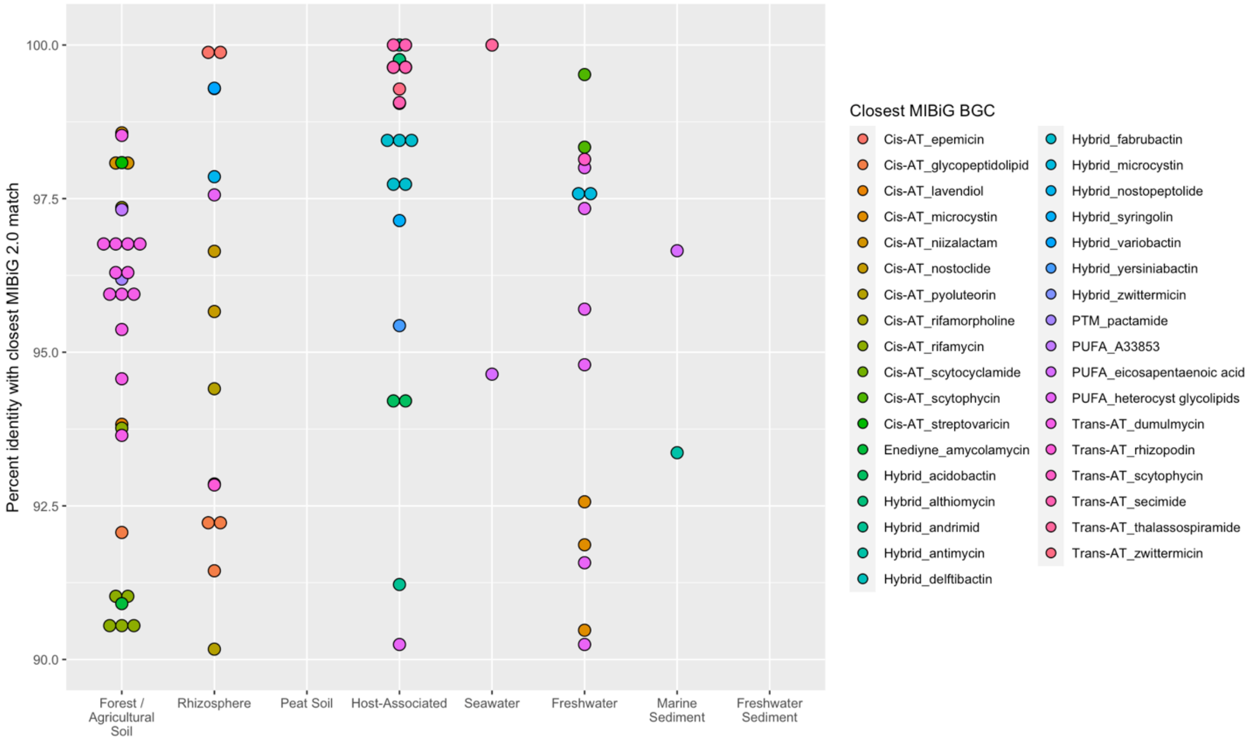

Figure S5 - Metagenomic KSs with ≥90% sequence identity matches with MIBiG 2.0. Dotplot indicates the metagenomic KSs with ≥90% sequence identity matches (y-axis) with sequences in the MIBiG 2.0 database. Matches shown by biome (x-axis) and colored by the NaPDoS2 classification. The legend indicates a compound produced by the closest matching MIBiG 2.0 BGC. The BGCs for microcystin, scytophycin, and zwittermicin have KSs belonging to multiple classes.

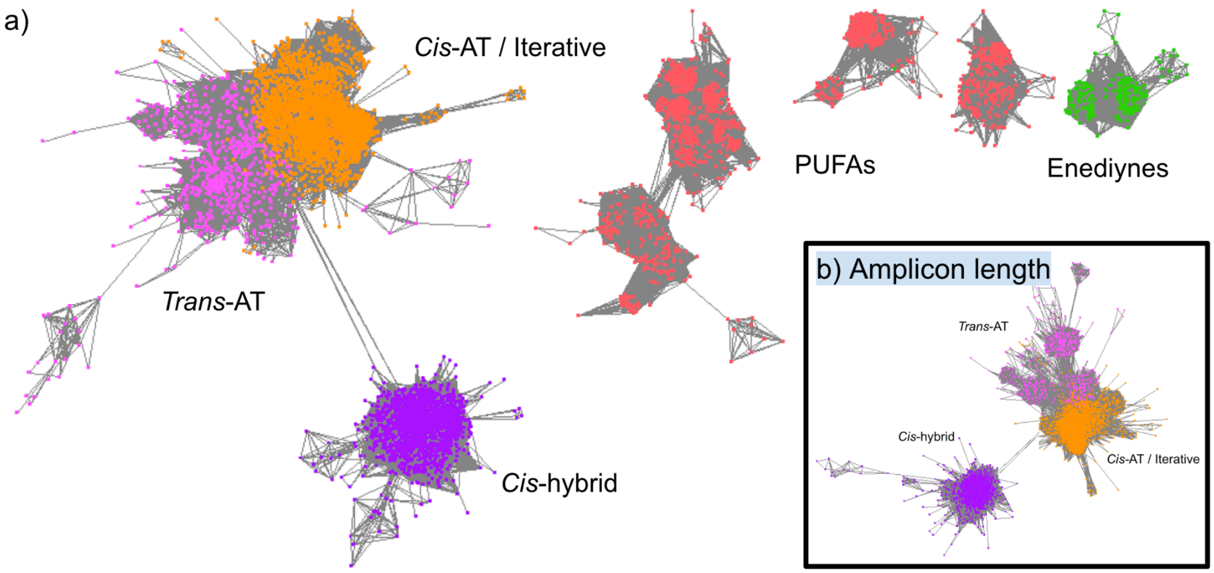

Figure S6 – Sequence similarity network of type I KS domains. A) SSN of full-length metagenome-extracted KS domains (amino acid sequences, average length = 420 aa) was constructed using EFI, visualized using Cytoscape, and colored according to NaPDoS2 classification revealing five major groups. The *cis*-AT/iterative group (orange) is a composite of KSs classified by NaPDoS2 as *cis*-AT modular, *cis*-loading, OLS, iterative aromatic, and iterative PTM. B) SSN created using the same methods after shortening the *cis*-AT/iterative, trans-AT, and cis-hybrid KSs to amplicon lengths (amino acids, average length = 138 aa) revealed similar clustering patterns as observed for the full-length KS sequences.

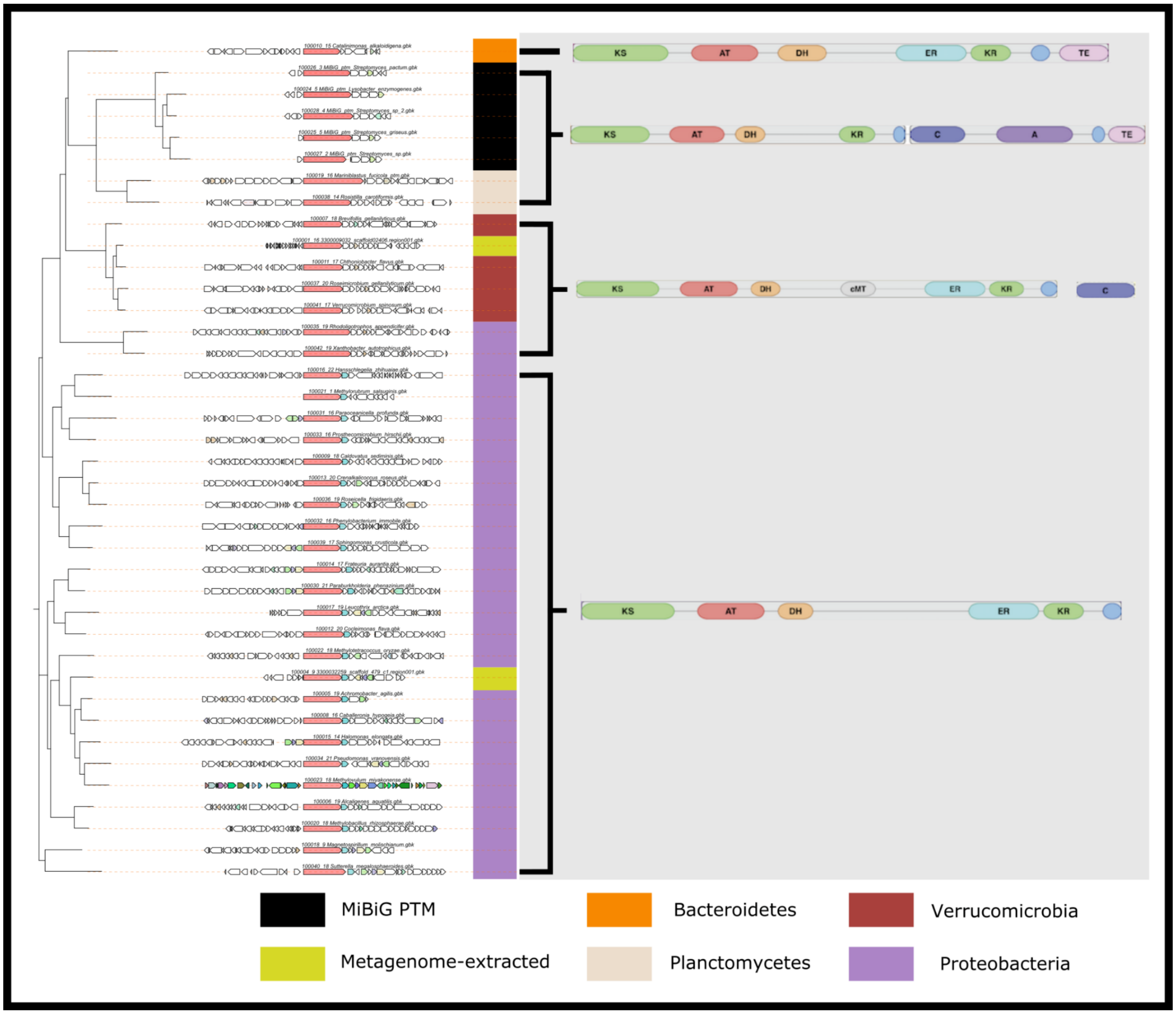

Figure S7 - Phylogeny and domain organization of the monomodular KS clade. Sequences within a major clade in the *cis*-AT/iterative KS phylogeny (Fig. 3a, pink) were classified as "iterative PTM" and thus predicted to be associated with monomodular PKS-NRPS genes. Two BGCs of sufficient length were identified in the metagenomes (yellow), analyzed using antiSMASH, and shown to possess PTM-like domain architectures in comparison to MIBiG reference BGCs (black) based on the domain organization of the core biosynthetic gene (pink). Related KSs were identified in RefSeq genomes (colored by taxa) based on NaPDoS2 KS classifications and a multi-locus BGC phylogeny used to show that the metagenome-extracted BGCs are most closely related to sequences observed in Verrucomicrobia and Proteobacteria. Subtle differences in the modular organization among these monomodular systems (brackets) suggests that new PTMs likely remain to be discovered.

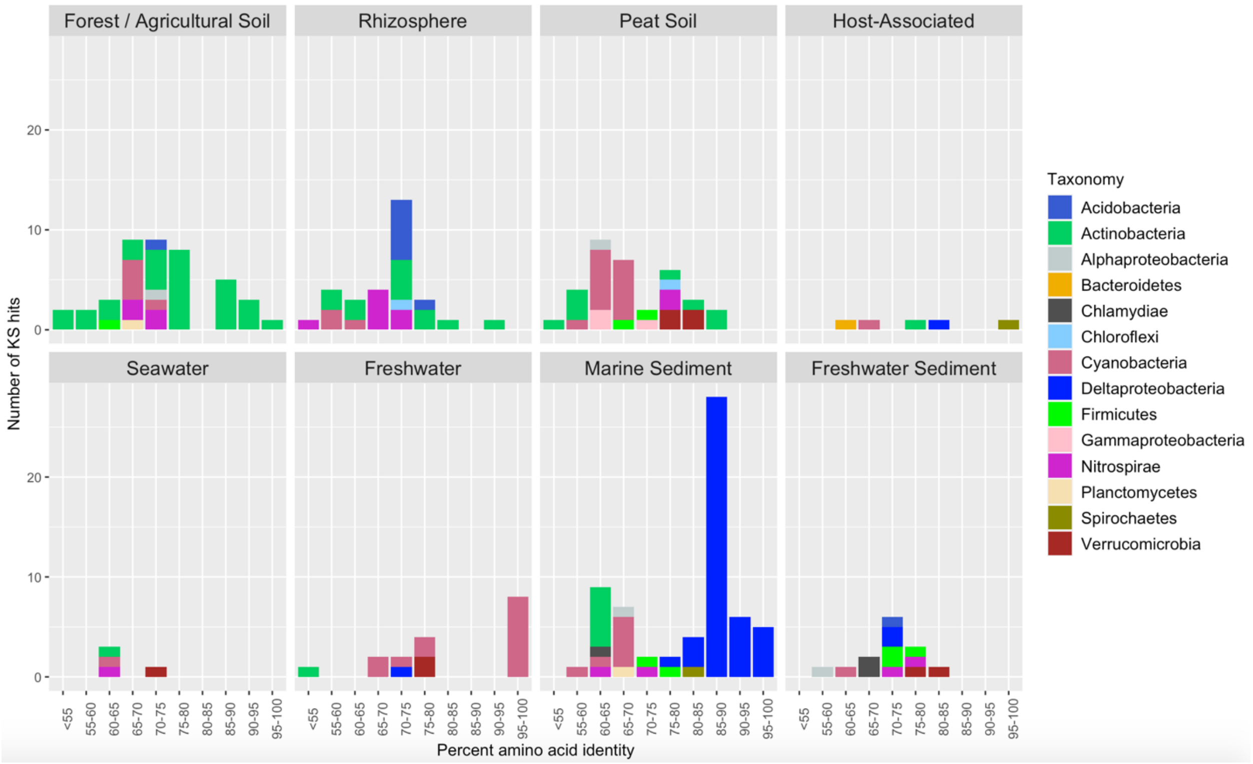

Figure S8- Enediyne KS domain distributions across biomes. Stacked bar charts indicate the phylum-level abundance (y-axis) of full-length metagenome-extracted enediyne KS domains across eight biomes grouped by percent identity with the closest NCBI BLASTp database match (x-axis).

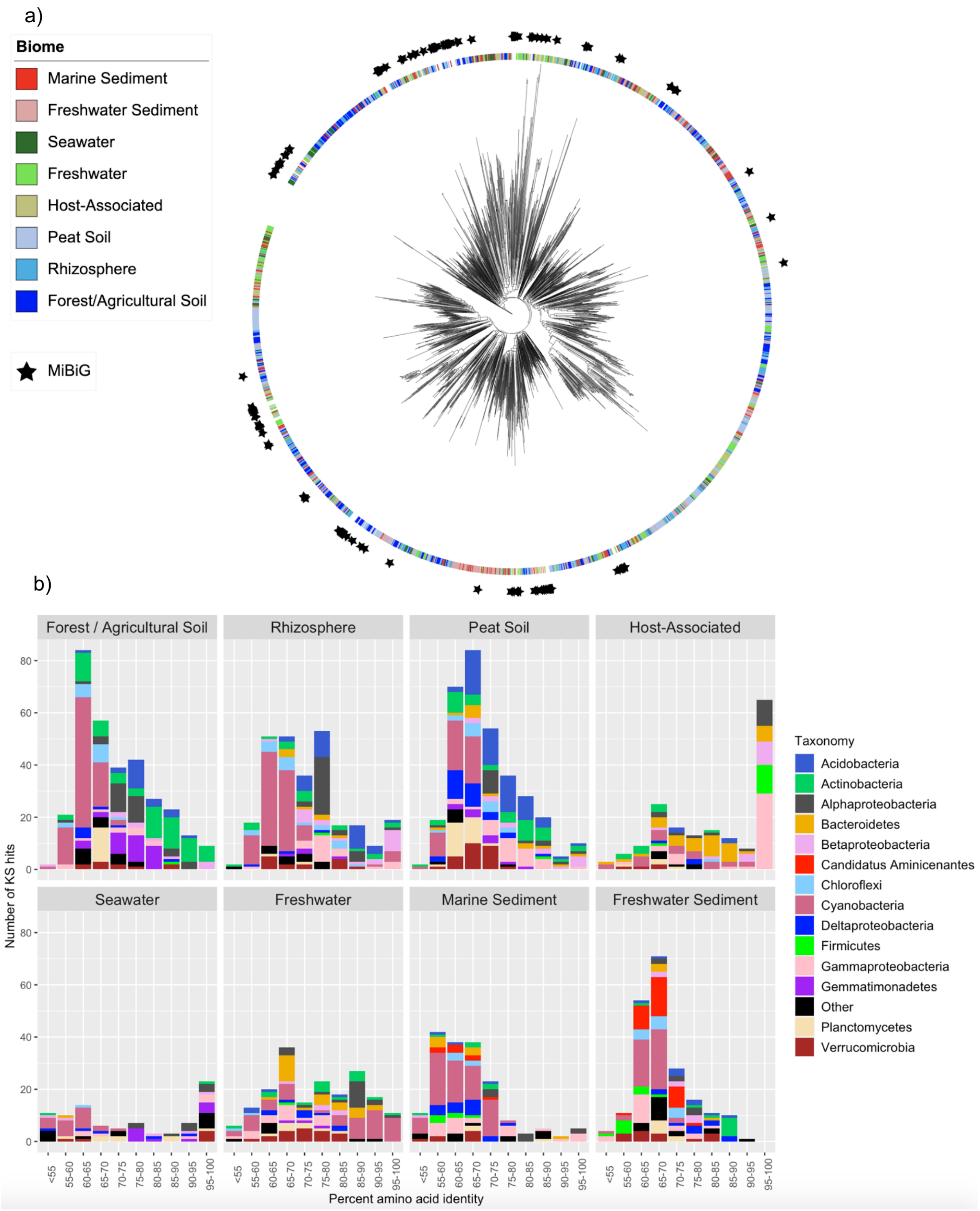

Figure S9 - *Cis*-hybrid KS domain phylogeny and distributions across biomes. A) FastME phylogeny generated from full-length metagenome-extracted *cis*-hybrid KS domains (n=1746, colored by biome) with the position of MIBiG-extracted *cis*-hybrid KS domains shown as black stars. B) Stacked bar charts indicate the phylum-level abundance (y-axis) of full-length

metagenome-extracted *cis*-AT KS domains across eight biomes grouped by percent identity with the closest NCBI BLASTp database match (x-axis).

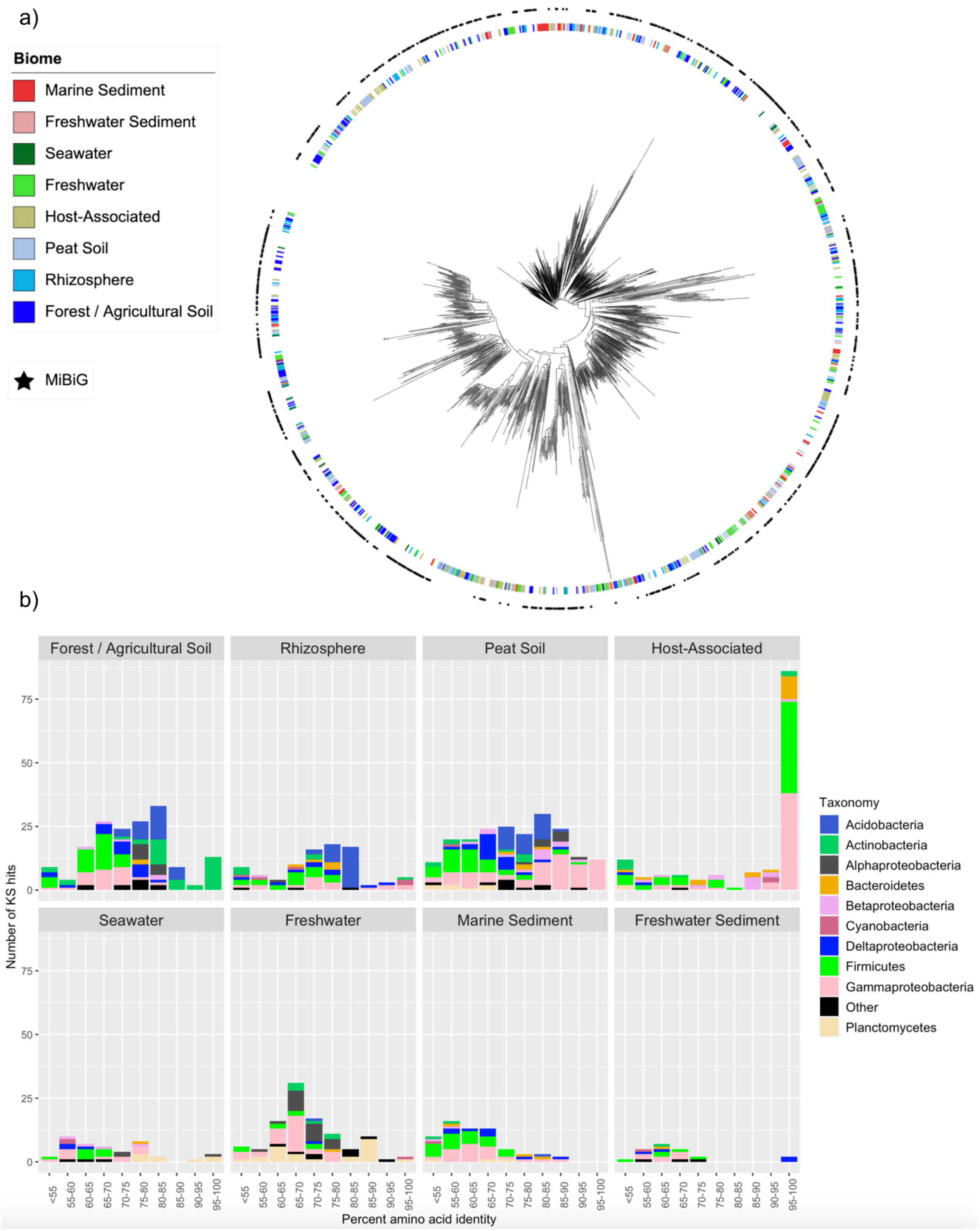

Figure S10 - *Trans*-AT KSs domain phylogeny and distributions across biomes. A) FastME phylogeny generated from full-length, metagenome-extracted *trans*-AT KS domains (n=831, colored by biome) with the position of MIBiG-extracted *trans*-AT KS domains shown as black stars. B) Stacked bar charts indicate the phylum-level abundance (y-axis) of full-length metagenome-extracted *trans*-AT KS domains across eight biomes grouped based on percent identity with the closest NCBI BLASTp database match (x-axis).

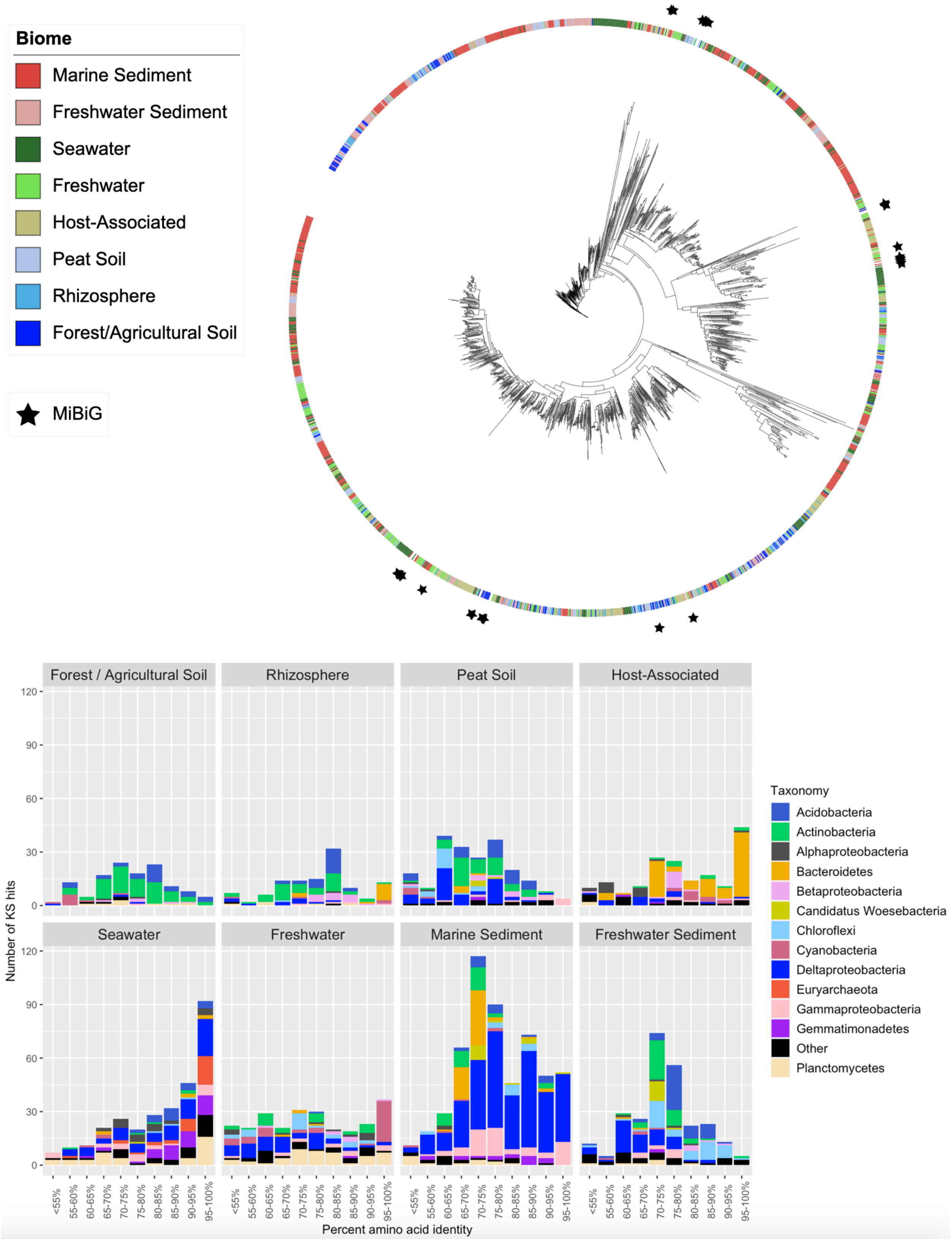
Biome
Marine Sediment
Freshwater Sediment
Seawater
Freshwater
Host-Associated
Peat Soil
Rhizosphere
Forest/Agricultural Soil
MiBiG
Forest / Agricultural Soil
Rhizosphere
Peat Soil
Host-Associated
Seawater
Freshwater
Marine Sediment
Freshwater Sediment
Number of KS hits
0
30
60
90
120
<55%
55-60%
60-65%
65-70%
70-75%
75-80%
80-85%
85-90%
90-95%
95-100%
Percent amino acid identity
Taxonomy
Acidobacteria
Actinobacteria
Alphaproteobacteria
Bacteroidetes
Betaproteobacteria
Candidatus Woesebacteria
Chloroflexi
Cyanobacteria
Deltaproteobacteria
Euryarchaeota
Gammaproteobacteria
Gemmatimonadetes
Other
Planctomycetes

Figure S11 - PUFA KS domain phylogeny and distributions across biomes. A) FastME phylogeny generated from full-length metagenome-extracted PUFA KS domains (n=1996, colored by biome) with the position of MIBiG PUFA KS domains shown as black stars. B) Stacked bar charts indicate the phylum-level abundance (y-axis) of full-length metagenome-extracted PUFA KS domains across eight biomes grouped based on percent identity with the closest NCBI BLASTp database match (x-axis).

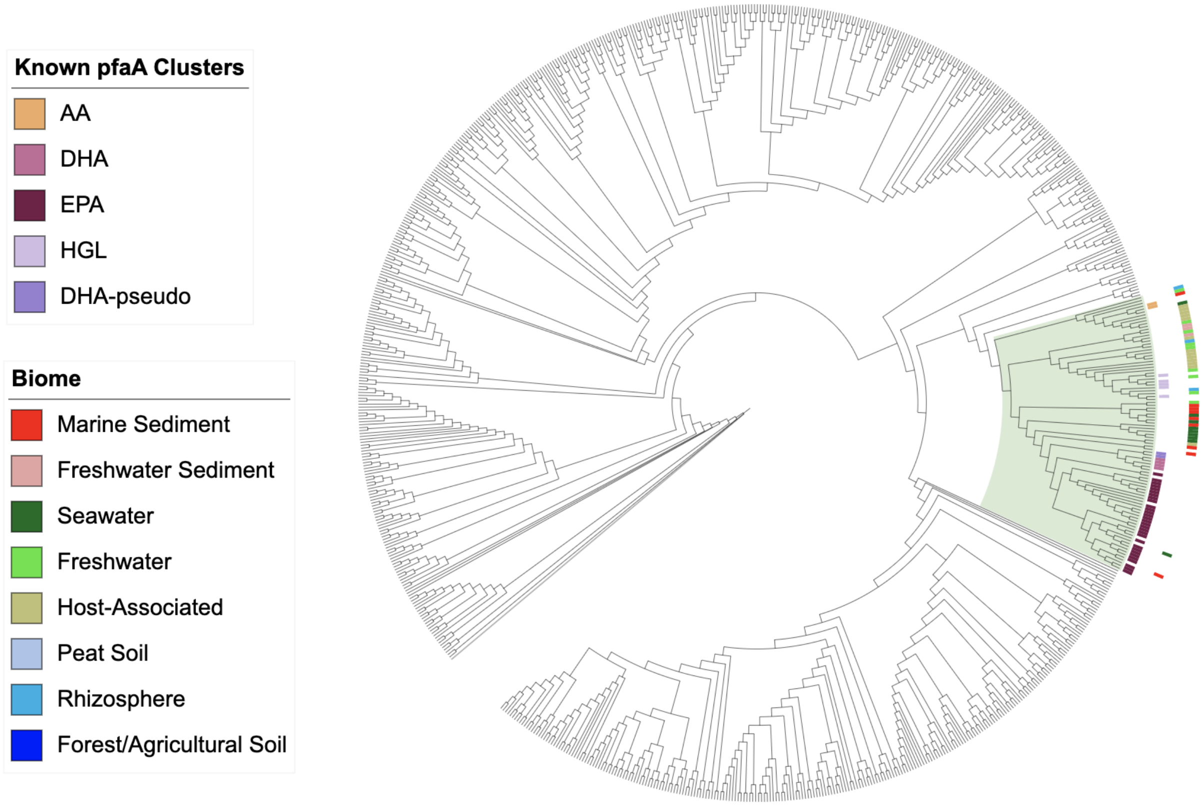

Figure S12 - pfaA KS domain phylogeny. FastME phylogeny generated from full-length metagenome extracted PUFA KS domains classified as pfaA (n=1170) compared with previously characterized pfaA BGCs (green highlight) associated with the production of arachidonic acid (AA), docosahexaenoic acid (DHA), eicosapentaenoic acid (EPA), heterocyst glycolipids (HGL), and pseudo-docosahexaenoic acid (pseudo-DHA) (1). Ninety-two percent of the metagenome extracted pfaA KS domains fall outside this clade.

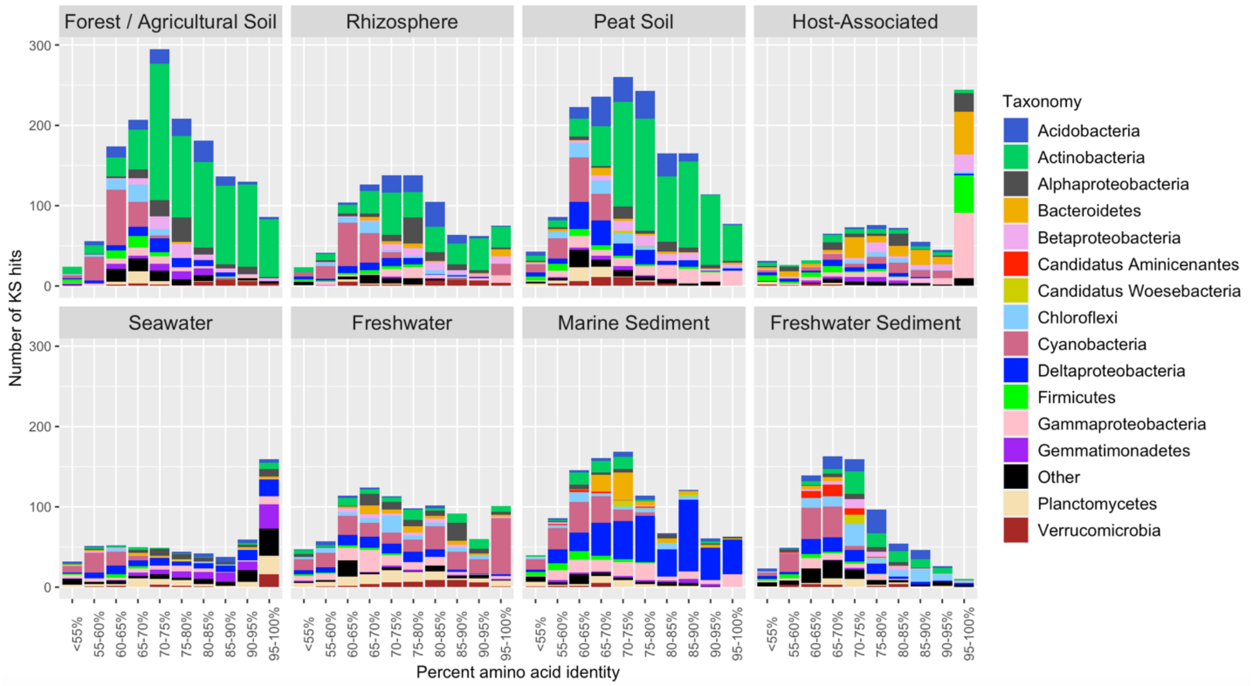

Figure S13 - Full-length KS domain distributions across biomes. Stacked bar charts indicate the phylum-level abundance (y-axis) of full-length metagenome-extracted KS domains across eight biomes grouped based on percent identity with the closest NCBI BLASTp database match (x-axis).

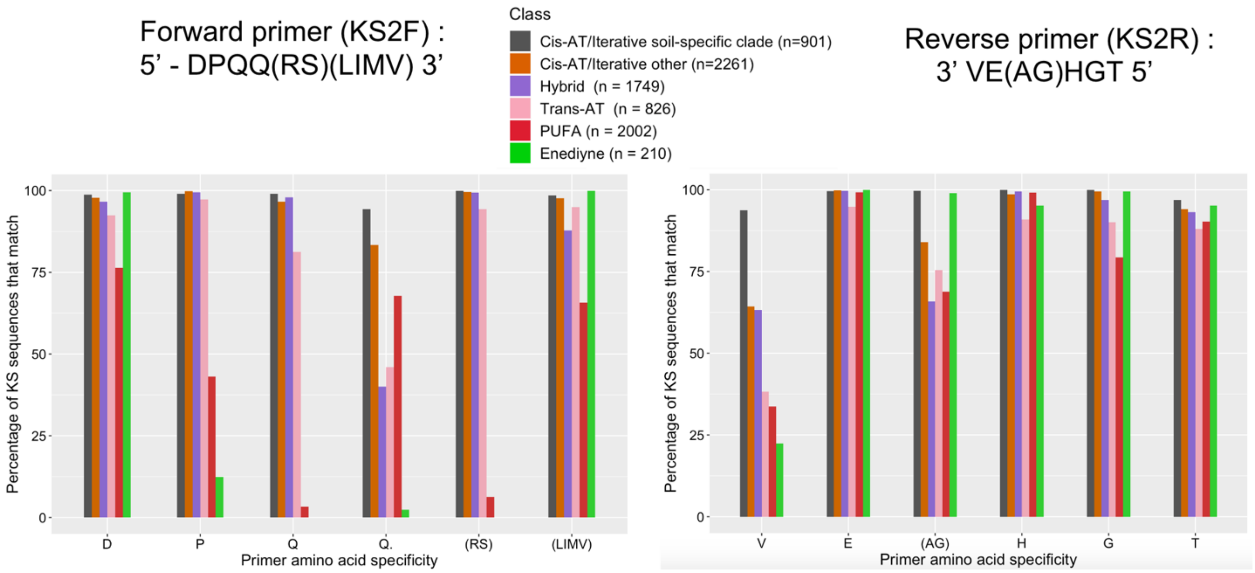

Figure S14 - Evaluation of the KS2F/R primer set. Percentage of metagenome-extracted KS amino acid sequences (y-axis) that match the KS2F/R primers at each position. The amino acid specificity of the primers is listed at each position by the one letter code with degeneracy indicated (x-axis). KS sequences are grouped by their NaPDoS2 classification. The *cis*-AT/iterative soil-dominant KSs (black) were analyzed separately from all other *cis*-AT/iterative KSs (orange).

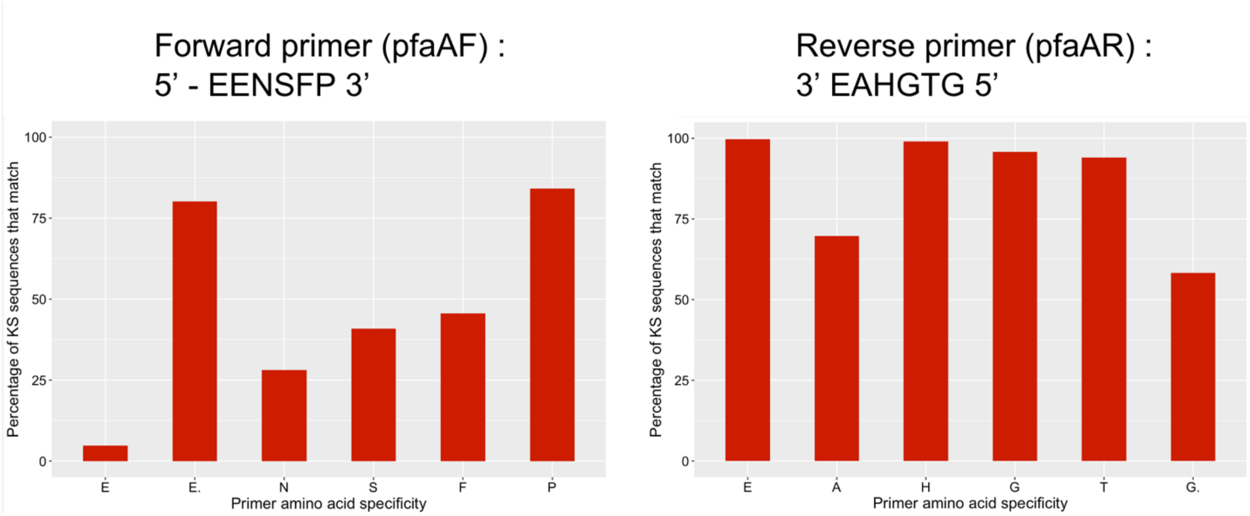

Figure S15 - Evaluation of the pfaA primer set. Percentage of metagenome-extracted KS sequences (amino acid format) classified as PUFA pfaA KSs by NaPDoS2 that match the pfaAF/AR primers at each position (y-axis). The amino acid specificity of the primers is listed at each position by the one letter code (x-axis).

References

1. Shulse CN, Allen EE. 2011. Diversity and distribution of microbial long-chain fatty acid biosynthetic genes in the marine environment. Environmental Microbiology 13:684-695.
